# A hypothesis for the pathogenesis of radiation-induced oral mucositis: when biological challenges exceed physiologic protective mechanisms. Implications for pharmacological prevention and treatment

**DOI:** 10.1007/s00520-021-06108-w

**Published:** 2021-03-13

**Authors:** Stephen T. Sonis

**Affiliations:** 1 Dana-Farber/Brigham and Women’s Cancer Center, Biomodels, LLC, Boston, MA USA; 2 Division of Oral Medicine, Brigham and Women’s Hospital, 75 Francis Street, Boston, MA 02115 USA

**Keywords:** Mucositis, Radiation, Pathobiology, Oxidative stress

## Abstract

Oral mucositis (OM) remains a significant unmet need for patients being treated with standard concomitant chemoradiation (CRT) regimens for head and neck cancers (HNC). OM’s pathogenesis is complex and includes both direct and indirect damage pathways. In this paper, the field is reviewed with emphasis on the initiating and sustaining role of oxidative stress on OM’s pathobiology. A hypothesis is presented which suggests that based on OM’s clinical and biological trajectory, mucosal damage is largely the consequence of cumulative CRT-induced biological changes overwhelming physiologic self-protective mechanisms. Furthermore, an individual’s ability to mount and maintain a protective response is dependent on interacting pathways which are primarily determined by a multiplex consisting of genomics, epigenomics, and microbiomics. Effective biologic or pharmacologic OM interventions are likely to supplement or stimulate existing physiologic damage-control mechanisms.

## Introduction

Radiation-induced toxicities (RIT) threaten treatment tolerance, cause tissue injury, compromise quality of life, and increase healthcare resource use [1]. No group is more at risk of RIT than head and neck cancer (HNC) patients. Even with improved radiotherapy (RT) technology and techniques, this cohort consistently suffers both acute and chronic RITs [2]. None is more impactful than OM.

Many past attempts to identify an effective pharmacologic solution for radiation-induced oral mucositis (ROM) have failed. It seems that underestimating the biological complexities of ROM’s pathogenesis contributed to missteps in accurately identifying druggable targets. Gone are the days when ROM was assumed to be solely due to direct DNA damage in epithelial stem cells, that a single group of cells (endothelium) influenced another (epithelium) [3], that normal and tumor cells responded identically to radiation, or that the molecular path leading to tissue damage was accurately represented by a linear cascade.

As the complexity of ROM pathogenesis has been further defined, we have mapped the steps leading to and sustaining injury, developed a biological event hierarchy, and sequentially integrated biological signaling into our picture of ROM’s clinical course. With this understanding, better strategies to identify effective druggable targets have ensued.

But executing on those strategies has been difficult. The biological cascade that follows a single acute dose of radiation is complicated. PubMed contains over 7000 papers describing normal tissue response to RT and about 10,000 with keywords including “radiation” and “normal tissue”. Overwhelmingly, each manuscript focuses on a single biological molecule, cell type, organelle, or pathway, at one time point. A global picture is absent. To put this into perspective, imagine you were tasked with drawing a country map by piecing together detailed maps of individual cities. Now imagine that the city maps changed daily; you get an idea of the difficulties in comprehensively defining ROM pathogenesis in the context of a fractionated radiotherapy course. A working paradigm describing ROM’s pathogenesis must address three observations. First, not only does radiation impact cancer cells and normal cells in different ways [4], but there is variability depending on normal cell type [5]. Second, while initial ROM clinical changes are attributable to immediate direct cell injury, its time and fraction-dependent progression and sustainment are dominated by indirect signaling, enzymatic shifts, and protein and non-protein intermediaries [6]. In aggregate, these indirect effects are the major contributors to ROM development. Finally, the extensive complex of biological events and mediators which initiate, catalyze, and sustain radiation-induced injury is dynamic. The degree with which it impacts ROM reflects the extent of homeostasis loss as a function of cumulative radiation dose. Thus, direct damage can be reasonably modeled as a radiation dose response, while the indirect damage pathway is subject to intrinsic genomic, epigenomic, metabolomic, and microbiomic [7] influences.

## Biological characterization of a moving target

**Fig. 1:**
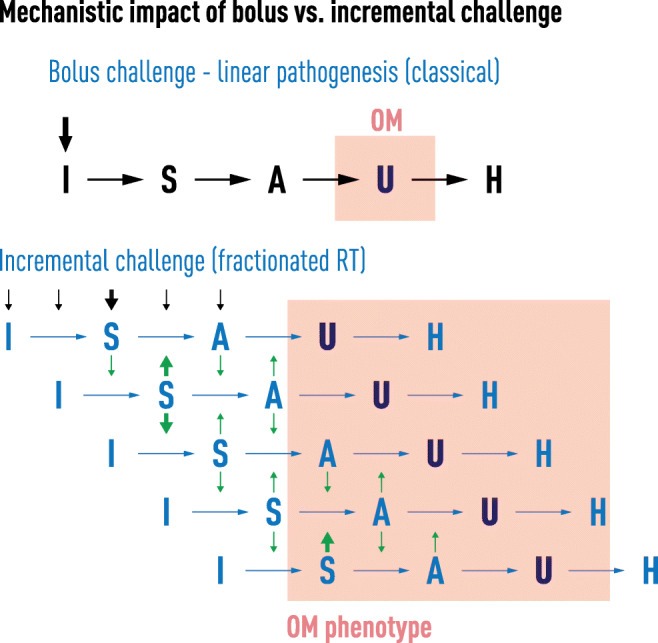
Differences in the clinical trajectory of oral mucositis between a bolus and fractionated stomatotoxic challenge: one blow from a sledgehammer vs. cumulative hits from a small ball peen hammer. The acuity of SOM development and resolution in patients receiving high-dose chemotherapy such as in conditioning regimens for stem cell transplants contrasts dramatically with the slower onset, duration, and time to resolution observed in patients receiving typical regimens of fractionated doses of radiation for the treatment of head and neck cancers. While there is similarity in the underlying pathogenic mechanisms, the repetitive challenge of multiple radiation doses produces a cumulative biological effect that results in injury, i.e., one blow from a sledgehammer vs. repeated hits with a small hammer

Contrasting with many models used to study radiation biology, clinical fractionated radiation regimens provide a different dosing dynamic and cell and tissue challenge. The biological “movie” following a single radiation exposure only partially reflects the repeated pounding suffered by normal mucosa during treatment in which the biological impact of radiation represents the sum of cumulative dosing. Further, the biological responses of previously irradiated cells and tissues will differ from those of radiation naïve ones, as is well recognized in the setting of clinical reirradiation. Whatever signals or pathways are induced one day are supplemented, overlapped, and modified on subsequent days of treatment (Fig. 1). Following radiation, lingering effects of treatment also project into delayed or late clinical events.

## Mucositis is dynamic morphologically

**Fig. 2:**
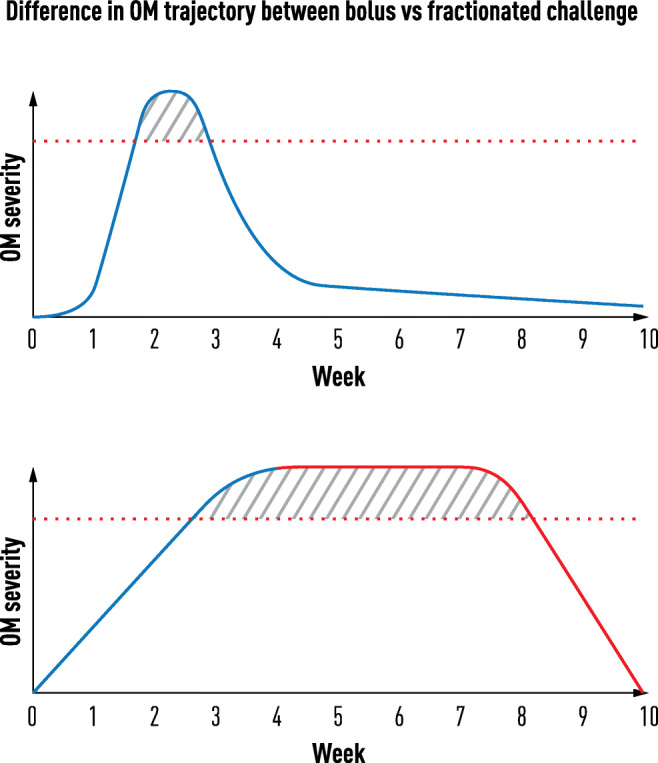
Fractionated radiated challenge influences biological interactions and phenotype in the development of SOM. The biological sequence which delineates the pathogenesis of mucositis has been described as a linear 5-phase process (5,6): initiation (I), signaling (S), amplification (A), ulceration (U), and healing (H). The concept was originally proposed based on data obtained following challenges with bolus chemotherapy in humans or a single dose of high energy radiation in animals (Panel 1). The sequence also characterizes the biological response and course associated with each daily fraction of radiation experienced by patients being treated for cancers of the head and neck. Critically, however, in cases of fractionated dosing, the net clinical effect is not only a consequence of the linear events initiated by each fraction but more importantly by the vertical crosstalk which recognizes that the biological consequences of each fraction not only affect tissue horizontally but also modifies or is modified by cell function primed by earlier doses (Panel 2)

The type of oral mucosa impacts ROM risk as it typically effects the movable or thin mucosa but spares the more keratinized tissue [8]. Mean turnover time for tissues at risk ranges from 14 days (buccal mucosa) to 20 days (floor of the mouth) [8]. Consequently, if a single radiation exposure sufficiently destroyed basal stem cells on day 0 to completely stop cell renewal, the phenotypic effect—ulceration—would be expected between 2 and 3 weeks later, precisely what is observed in patients treated with bolus doses of stomatotoxic chemotherapy (Fig. 2) [9]. This contrasts with the extended onset and course of ROM where small incremental RT fractions are administered over time [10].

ROM’s clinical trajectory is well-known [10]. Most patients receiving 2 Gy daily fractions manifest clinical evidence of reduced renewal (epithelial atrophy) about 2–3 weeks after radiation start and peak ulceration by 4–5 weeks. In the context of mean turnover time, this would suggest that a cumulative radiation dose of about 20–25 Gy is necessary to overcome physiologic defense mechanisms and irreversibly injure enough basal stem cells to tip the balance from maintenance of homeostasis to generation of ulcerative ROM (Fig. 2).

Ulceration heralds the start of a biological tug-of-war in which cell-signaling pathways favoring wound healing are triggered but face an ongoing bombardment of biological drivers of ROM, including additional radiation fractions, reactive oxygen species (ROS), and NF-κB-driven (nuclear factor-kappa B) inflammation.

## Biological concepts: ROM and dominos

ROM pathogenesis consists of parallel, sequential, and staggered molecular events occurring in a temporal dimension. Were ROM simply the consequence of a series of interdependent occurrences, it might be modeled by a row of dominos where each domino represented a component in the pathogenetic path. Hit the first domino and the next domino falls, then the next, and so on until ulceration occurs. However, such a linear representation ignores the complicated relationships which characterize ROM’s biological processes. A more realistic arrangement would account for the intricacies, redundancies, and interactions that occur, perhaps a pyramid arrangement in which the first domino hits the next 2, those hit the 4 behind them, etc. But that still would not accurately reflect biological dynamics which might require that 2 of the dominos in row 10 could not only fall backward, but also sideways, or that a looping row of dominos could project from the major group and circle back to re-knock over recovered dominos. Even this model fails to recognize that the size and weight of each domino might vary from patient to patient (genomic variability), that the space between dominos is not the same (epigenetics), or even that the force applied to each is not equivalent (amplifying potential of the microbiome).

## An alternative concept of ROM pathogenesis

A hypothetical argument suggests that ROM pathogenesis describes the cumulative effect of two distinct pathways which occur in a semi-staggered sequence. The first pathway, the immediate injury pathway, induces direct damage to basal stem cells, whereas injury induced by the second pathway*,* the indirect pathway, reflects the failure of normal host defense mechanisms to keep up with biological challenges imparted by accumulating radiation doses. The two occur in parallel, but their contribution to ROM pathogenesis is staggered and not equivalent. Within and between each pathway are interactions between cells, their components, signaling molecules, transcription factors and their products, and positive and negative feedback loops. While basal epithelial cells are the target end organ, inflammatory and non-inflammatory cells in the submucosa are active participants serving as message generators and carriers, both during the active period of radiation therapy and as conduits for late tissue changes [11]. And the tissue environment is dynamic: Mucosa irradiated on day 1 is not the same days or weeks later after being conditioned by chronic radiation exposure. Once gap junctions are disrupted, the microbiome also becomes a participant. This all occurs in the context of patients’ baseline physiological state which is likely altered by biological activity emanating from the tumor response to irradiation [12].

The concept of radiation injury being mediated by direct and indirect mechanisms has been described [13] (Hall S et al. as an example). What is novel is the idea that trajectory and severity of RIT are driven by an irrevocable tipping point in the balance between radiation-associated damage mechanisms and the capacity of physiological protective mechanisms to meet that challenge.

The immediate injury pathway causes basal epithelial cell death in the absence of intermediaries primarily through direct ionization of DNA chemical bonds and DNA cleavage by hydroxyl radical from ionization of water, both generating radiation-induced DNA double-strand breaks (DSB) [14]. While DSBs only have an identifiable role in 30% of radiation injuries [15], damage occurs quickly and primes subsequent downstream events. The immediate pathway is responsive to radiation dose and schedule [16], but not markedly impacted by intrinsic response modifiers such as genetics. The early phenotypic consequences of ROM are most likely attributable to the immediate pathway. The threat of the immediate pathway as the sole provocateur of ROM is less than that of the indirect pathway as suggested by the finding that the 3000 DNA lesions per cell noted after the first exposure to a 2 Gy fraction [17] is well within the ability of cell repair and is far less than the number of breaks associated with normal oxygen metabolism.

In contrast, the indirect pathway is responsible for most of the acute and chronic tissue effects associated with ROM. Its activity is reflective of the inability of physiologic defense mechanisms to mitigate accumulating radiation stressors resulting in an imbalance in which biological injury drivers such as free radicals and damaging cytokines cannot be sufficiently controlled as other damage signals are initiated. Those mechanisms that provide protection and healing are superseded by a cascade of destructive elements. Primary cytoprotective actions are replaced by the biological imperative to eliminate irreversibly damaged cells or mutated DNA. Whereas the immediate pathway is uninterrupted and focused, the indirect pathway is more nuanced and contains several different mediators, most under genetic control, which interact with each other. It is, therefore, sensitive to patients’ genomics, tumor biology, and other intrinsic factors. Its impact becomes most clinically obvious when the threshold of a patient’s cytoprotective mechanisms is surpassed. Ultimately patients’ risk and course of ROM reflect the cumulative effect of both immediate and indirect pathways.

Overwhelmingly, it is injury provoked by non-DSB mechanisms that accounts for most RITs [18]. The DNA damage response results in ATM-mediated p53 activation [19], lipid peroxidation, membrane damage [20], and sphingomyelinase activation [21] and prompts activation of the innate immune response [22,23] and inflammasome [24]. But it is uncontrolled oxidative stress that plays the biggest role in the indirect pathway [20], leading to the greatest turmoil which ultimately poses the most significant mucosal survival threat. Couple that with an inability of cell repair processes to keep pace with ROS-induced injury and the balance tips in favor of severe ROM.

## Normal mechanisms that control oxidative stress are ultimately overwhelmed

Seventy percent of radiation-induced cell injury is attributable to oxidative stress [15]. Normally, cells maintain a level of redox homeostasis by interacting enzymatic and non-enzymatic mechanisms [23]. RT induces ROS formation with such ferocity that normal protective mechanisms are overwhelmed. Excessive ROS levels directly disrupt cells and further serve as secondary messengers to elicit responses, resulting in acute and chronic normal tissue injury through ligand/receptor-initiated pathways including MAPK, pI3K, and NF-κB [23,25]. By interfering with transcription factor activation through redox sensitive cysteine residues in DNA-binding sites, ROS affects such critical pathways as those associated with NF-κB, AP-1, and hypoxia-inducible factor (HIF-1α) [23]. There is continuous dynamic interaction and crosstalk which becomes increasingly multifarious as their biological downstream consequences become evident. Fundamentally then, it is the insufficient capacity of normally functioning redox control systems that dominates radiation-induced normal cell destruction.

Control of oxidative stress is so critical to cell survival that redundant processes contribute to its management at two levels: systems (largely enzymatic) that process certain ROS to make them non-pathogenic and damage-control mechanisms which amplify antioxidant mechanisms.

Cells are 70% water, and this plays a key role in the oxidative stress from radiation. Radiolysis of water hydroxyl radical (^●^OH) directly cleaves tumor DNA. It also initiates the formation of superoxide (O_2_^●-^), a major cause of oxidative stress, and uncontrolled, the first indirect pathway blow to normal cell survival [26]. Ionizing radiation further activates cell wall NADPH oxidases generating even greater amounts of O_2_^●-^ in the hours following treatment. Later in the indirect pathway, inflammatory cells recruited to the sites of emerging tissue injury may produce O_2_^●-^ for weeks after irradiation. In response to escalating O_2_^●-^, the cell counters with “first line antioxidants” [27] a network of enzymes, including superoxide dismutases (SODs), catalase, and glutathione peroxidase, of which the SODs play a lead and anchoring role [28].

Enzymatic degradation of superoxide occurs in two steps: first the conversion of O_2_^●-^ to hydrogen peroxide (H_2_O_2_) and molecular oxygen (O_2_), which is mediated by SODs, and second, conversion of H_2_O_2_ to water and O_2_ by catalase and glutathione peroxidase:

$$ {{\mathrm{O}}_2}^{\bullet -}\to {\mathrm{H}}_2{\mathrm{O}}_2\to {\mathrm{H}}_2\mathrm{O}+{\mathrm{O}}_2 $$

To facilitate degradation of O_2_^●-^, three SOD enzymes are present in human cells: SOD1 (Cu/ZnSOD) in cytoplasm, SOD2 (MnSOD) in mitochondria, and SOD3 (EcSOD) in the extracellular space [29,30]. MnSOD is the most significant in maintaining redox homeostasis and having a protective role in response to RT [31] as evidenced by the observation that its absence is incompatible with life [32]. Redox homeostasis is critical to cell survival, so signaling and regulatory pathways that target antioxidant enzyme function are highly impactful, especially during the early stages of RT when they play a critical role in delaying injury. In the context of ionizing radiation, MnSOD expression, activation, and function are influenced by at least four pathways: a direct response to superoxide formation [33], a consequence of NF-κB activation via innate immune response [23, 34], through mTOR signaling [35], and in response to stimulatory signaling through p53 [29].

Simultaneous with intracellular O_2_^●-^ induction prompting an immediate MnSOD response, activation of the innate immune response, and inflammasome occur as cells struggle to cope with radiation’s toxic effects [14,36]. DSBs are particularly effective in eliciting acute cell injury resulting in apoptosis or necrosis [15]. Nuclear molecules passively released from damaged cells start the process of activating a non-pathogen based innate immune response. Alarmins, notably HMGB1, a damage-associated molecular pattern molecule (DAMP), bind to pattern recognition receptors (PPR) such as members of the toll-like receptor group to stimulate phosphorylation of cytoplasmic IKK and activate NF-κB [37]. Activation of the NLRP3 (NLR family pyrin domain containing 3) inflammasome is catalyzed, not only by DAMPs and PAMPs (pathogen-associated molecular pattern) [38] but by O_2_^●-^ itself [39].

NF-κB activation results in the expression of many target genes, including the transcription of MnSOD to enhance mitochondrial antioxidation [32,40,41]. As a signaling molecule, H_2_O_2_ also provides supplemental NF-κB activation [42]. In contrast to O2^●-^, H_2_O_2_ is stable and non-polar, so it easily passes through membranes, enabling signaling functions distant from the source and enhancing its ability as a messenger for some cell types [43].

The relationship between ROS and NF-κB has been reviewed [34]. NF-κB has been largely associated with cell survival, and it is in that context that it mitigates ROS-induced apoptosis or necrosis by activation of target genes which impact ROS production [23]. Interaction and crosstalk between ROS and NF-κB-JNK to prevent sustained JNK activation impact p53 [44] (see below) and thus favorably affect survival.

NF-κB’s central role in RIT pathogenesis is well-established [45]. Many NF-κB-associated pro-inflammatory cytokines track with RIT severity [6]. And relationships between overexpression of cytokine genes has been linked to OM risk [46]. Furthermore, cytokine-mediated messaging plays a role in feedback loops which amplify steps leading to injury [40]. Thus, the consequences of NF-κB likely change in response to the accumulation of radiation stress. Cell adhesion, acute phase proteins, stress response, and regulators of cell death and survival are all within its functional catchment and pro-inflammatory cytokines in perpetuating oxidative stress [47].

## Help is on the way (or is toxicity inevitable?)

An impressive example of biological crosstalk in radiation response is the interaction between MnSODs and the Keap1-Nrf2 (nuclear factor erythroid-2-like) pathway [48]. Nrf2 is a key transcription factor in maintaining redox homeostasis and appears to be a noteworthy contributor to host radiation response [49]. Interestingly, two aspects of Nrf2 behavior align with clinical observations associated with ROM. First, Nrf2 activity declines with age and thus is consistent with RIT risk [50]. Second, in agreement with clinical observations of OM behavior, Nrf2 activity varies with circadian rhythm [51]. While concordance between Nrf2 and ROM has not been specifically studied, ROM risk based on time of day of radiation has been reported [52].

Under normal conditions, the Keap1-Nrf2 complex inhabits the cytoplasm, with Keap1 keeping Nrf2 in check. Under significant stress or in response to signaling, Nrf2 is released from its Keap constraint and migrates to the nucleus where it is activated to control the transcription of over 400 genes [53]. Functionally, Nrf2 binds to and regulates the expression of acute radiation expression (ARE) genes which encode for both antioxidant proteins, including SODs, and a second tier of antioxidant enzymes including glutathione transferase and metallothioneins [54].

MnSOD signaling catalyzes Nrf2 upregulation [48]. In MnSOD-silenced hepatocytes, MnSOD acted as a signaling mediator for Nrf2-related survival genes and that expression of Nrf2 and its nuclear translocation could be mediated by MnSOD signaling [48].

In a reciprocal mechanism that might be interpreted as a cellular attempt to accelerate the control of potentially toxic levels of ROS, ARE-bound Nrf2 activates MnSOD [55] in a manner that is consistent with the importance of redox sensitivity in regulating Nrf2 and NF-κB in the genesis of ROM [56]. Furthermore, not only are reductions in Nrf2 associated with a parallel response in SODs, but HO-1, a Nrf2 target gene, is linked to Nrf2’s ability to attenuate NF-κB expression [57].

Of interest relative to the clinical trajectory of developing ROM is the finding that Nrf2 activation is radiation dose dependent. Activation has been noted at radiation fractions of 2 Gy [58]. However, unlike the first line enzymatic response which is triggered by NF-κB and AP1 within minutes or hours after the first dose of irradiation, Nrf2 antioxidant induction and response are slower. Reportedly, there is a 5-day delay before the Nrf2 antioxidant activation is observed. Possibly, the Keap1-Nrf2 pathway serves as a lifeboat against threatening levels of oxidative stress that have thwarted effective control by first line enzymes.

## It is not just the nucleus that is targeted by the immediate and indirect pathways

The impact of radiation on membrane lipids also contributes to the trajectory of cell injury, although the extent and timing are not well established [59]. A membrane stress apoptotic pathway has been described in which lipid peroxidation impacts both cell and mitochondrial membranes [60]. In the case of the cell membrane, the key messenger mediating apoptosis is ceramide. DSBs induced by high cumulative doses of radiation can directly activate ceramide synthase with the consequent generation of ceramide. Additionally, the effect of ROS, including O_2_^●-^, leads to lipid peroxidation, sphingomyelinase activation, and hydrolysis of membrane sphingomyelin to yield ceramide. In both cases, ceramide targets the RAC1/MAPK pathway which leads to the expression of MAPK8 and caspases 1, 3, and 6 and stimulation of the autocrine death receptor pathway. The significance of MAPK8 is noteworthy as it provides another interactional mechanistic feature given its implication in TNF-mediated apoptosis [61].

Mitochondrial membranes are also impacted as they respond to radiation-induced cytochrome c, activate the caspase cascade, release Ca++, and produce pro-apoptotic proteins [62]. The lipid peroxidation noted in mitochondria is associated with an increase in membrane permeability [62] which may signal the organelle’s demise.

## P53—tipping the balance?

Radiation-induced DNA strand breaks initiate a p53 DNA-damage response [19]. Triggered by ATM activation, p53 accumulates in the nucleus to initiate cell cycle arrest and promote survival and regulation of oxidative stress and the intrinsic apoptosis pathway [63]. P53’s impact on the cell’s response to oxidative stress varies with the level of ROS. During the initial stages of radiation, when ROS is modest, p53 signaling favors antioxidation by increasing antioxidant enzyme transcription including MnSOD [64]. However, once the extent of oxidative stress outstrips the ability of cytoprotective mechanisms to cope with increasing ROS levels as a consequence of cumulative radiation, the role of p53 shifts from promoting cell survival to protecting the organism from irrevocably damaged DNA. JNK signaling activates pro-oxidant genes such as p53-upregulated modulator of apoptosis (PUMA). Lipid peroxidation is enhanced and previously supported MnSOD activation is instead impaired through mitochondrial disruption [20].

## Clinical implications: how does the complexity of toxicity pathogenesis impact risk assessment, druggable targets, and precision medicine?

ROM occurs in two phases. Immediate injury mediated primarily by DSBs begins right after the first dose of radiation. Manifestations of cell injury such as ROM are, at this stage, probably limited and reversible up to a threshold dose, perhaps around 30 Gy. Mechanisms associated with immediate injury are only modestly influenced by, or responsive to, intrinsic factors such as patient genomics.

The indirect pathway is, for normal tissue, catastrophic, most clinically meaningful, and the result of cytoprotective mechanisms being overwhelmed by radiation-induced stress. It occurs at a threshold dose of radiation at which the cell and tissue capacity to neutralize or reverse the physiologic impact of recurrent radiation challenges is surpassed. The indirect pathway differs from the immediate pathway in three major ways: First, the radiation threshold dose at which it is initiated varies among patients as it is the subject to intrinsic controlling mechanisms including genomics, metabolomics, epigenomics, and microbiomics; second, it is indirect and enabled by intermediates including mediators of oxidative stress, cytokines, and enzymes; and third, it is influenced by signaling from bystander cells [65].

**Fig. 3:**
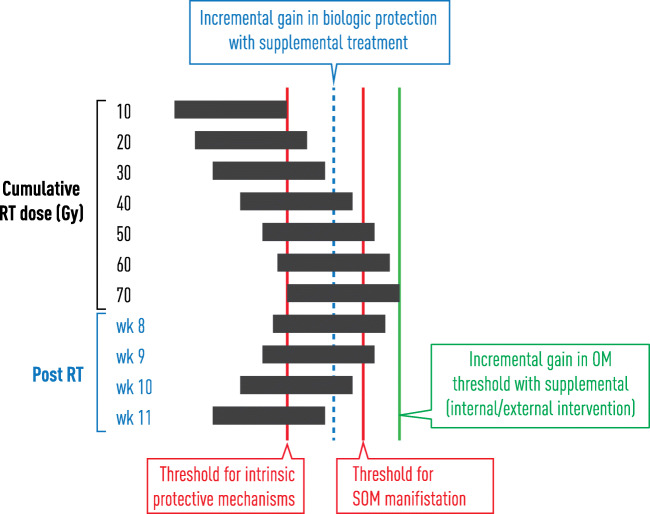
Mucositis is the consequence of the cumulative biological effects of the radiation challenge exceeding the intrinsic capacity of physiological protective mechanisms. Increasing the threshold for a gain in biological protection results in consequent increase in the threshold for manifestations of mucosal injury. Interventional strategies that supplement protective mechanisms and pathways or those that stimulate intrinsic mechanisms to optimize their effectiveness have proven to be most effective.

The consequences of these differences provide insight into clinical behaviors, opportunities for ROM risk prediction, and intervention targeting (Fig. 3). From a hierarchical standpoint, the ability to contain ROS and blunt the downstream effects of key transcription factors seems an obvious strategy for interfering with the indirect pathway. In the face of out-of-control ROS levels, solely blocking downstream events is likely to fail. So this leads to four approaches: (1) reducing oxidative stress by scavenging free radicals; (2) combination agents which are pleiotropic, effectively mitigating ROS levels, transcription factor activation, and cytokine production; (3) extrinsic supplementation in which therapeutically administered “lifeboat” compounds metabolize excessive ROS; and (4) pharmacologically mediated expression or activation of ROS-controlling enzymes.

There is evidence that all four strategies have merit. A scavenger, amifostine, was a product of an early US Army’s Antiradiation Drug Development Program. Its clinical potential was realized when in 1999 amifostine was approved as a salivary gland protectant from RT. Although amifostine’s mechanism of action has been primarily attributed to free radical scavenging, it also appears to act in the p53 pathway [66]. Amifostine’s effectiveness as an ROM intervention is unclear with conflicting trial results. Palifermin (KGF1) showed moderate effectiveness in reducing severe ROM incidence [67,68]; however, its use in solid tumors has been largely proscribed because of its potential impact on KGF-1 receptor-bearing cancer cells. Palifermin is biologically pleiotropic: Aside from its stimulating epithelial proliferation, it interacts with a range of mechanisms associated with RIT including NF-κB and Nrf2 [69].

SODs play a major role in mitigating radiation-induced ROS-mediated signaling and damage [29,30,51,70,71]. In 1987, radioprotection was observed in mice treated with intravenously administered SOD [72]. Soon after, SOD1 (orgotein) was shown to be clinically effective in treating RITs associated with head and neck radiation [73] and late toxicities in patients receiving pelvic irradiation [74]. Gene therapy using SOD2 plasmid/liposomes also protected mice from radiation-induced esophagitis [75]. More recently, a SOD mimetic (avasopasem manganese) effectively reduced the incidence and duration of severe ROM in patients being treated for oral and oropharyngeal cancers [76]. Avasopasem is currently in a large phase 3 ROM trial (NCT03689712) and a smaller phase 2 trial for radiation esophagitis (NCT04225026).

The final approach aims to optimize internal ROS defenses by provoking Nrf2 activation [77–80]. While ROS intervention has demonstrable importance as an interventional target, other related targets early in the ROM biological cascade (innate immune modifiers and NF-κB) may also be impactful [81, 82].

## Conclusion

RITs such as ROM have two distinct, but interactive pathways, immediate/direct and indirect. An effective management strategy must consider both optimal drug targets and dosing schedules. A treatment which maximally attenuates the indirect pathway and acts at a key “chokepoint” is likely to favorably impact the severity, incidence, and course of ROM without negatively impacting tumor response.

There is no FDA approved drug therapy to mitigate ROM in HNC patients. RITs, including ROM, remain common, create suffering for patients, threaten optimum cancer therapy, drain healthcare resources, and share pathogenesis components and sequences. Thus, a mechanistic understanding of ROM’s pathogenesis is critical to successful development of therapeutic interventions.

## Data Availability

N/A.

## Code availability

N/A.

## References

[1] Lalla RV et al (2019) Oral mucositis due to high-dose chemotherapy and/or head and neck radiation therapy. J Natl Cancer Inst Monogr 2019(53):Igz011 10.1093/jncimonographs/lgz011 31425601

[2] Maria OM (2017). Radiation-induced oral mucositis. Front Oncol.

[3] Bowen J, Al-Dasooqi N, Bossi P (2019). The pathogenesis of mucositis: updated perspectives and emerging targets. Support Care Cancer.

[4] Hongsheng Y (2013). Different responses of tumor and normal cells to low-dose radiation. Contemp Oncol (Pozn).

[5] Wodarz D, Sorace R, Komarova NL (2014). Dynamics of cellular responses to radiation. PLoS Comput Biol.

[6] Citrin D, Mitchell JB (2017). Mechanisms of normal tissue injury from irradiation. Semin Radiat Oncol.

[7] Kumagi T (2018). The microbiome and radiation-induced bowel injury. Evidence for potential mechanistic role in disease pathogenesis. Nutrients.

[8] Squier CA, Kremer MJ (2001). Biology of the oral mucosa and esophagus. J Natl Cancer Inst Monogr.

[9] Spielberger R, Stiff P, Bensinger W, Gentile T, Weisdorf D, Kewalramani T, Shea T, Yanovich S, Hansen K, Noga S, McCarty J, LeMaistre CF, Sung EC, Blazar BR, Elhardt D, Chen MG, Emmanouilides C (2004). Palifermin for oral mucositis after intensive therapy for hematologic cancers. N Engl J Med.

[10] Sonis ST (2009). Mucositis: The impact, biology and therapeutic opportunities of oral mucositis. Oral Oncol.

[11] Fajardo LF (2005) The pathology of ionizing radiation as defined by morphologic patterns. Acta Oncol 44:13–22 10.1080/02841860510007440 15848902

[12] Ungefroren H, Sebens S, Seidl D, Lehnert H, Hass R (2011). Interaction of tumor cells with the microenvironment. Cell Commun Signal.

[13] Hall S, Rudrawar S, Zunk M, Bernaitis N, Arora D, McDermott C, Anoopkumar-Dukie S (2016). Protection against radiotherapy-induced toxicity. Antioxidants.

[14] Giuranno L (2019). Radiation-induced lung injury (RILI). Front Oncol.

[15] Santivasi WL, Xia F (2014). Ionizing radiation-induced DNA damage response and repair. Antioxid Redox Signal.

[16] Nickoloff JA (2018). Translational research in radiation-induced DNA damage signaling and repair. Transl Cancer Res.

[17] Lomax ME, Folkes LK, O'Neill P (2013). Biological consequences of radiation-induced DNA damage relevance to radiotherapy. Clin Oncol.

[18] Russi EG et al (2014) Local and systemic pathogenesis and consequence of regimen-induced inflammatory responses in patients with head and neck cancer receiving chemoradiation. Mediators Inflamm 2014:518261. 10.1155/2014/518261 10.1155/2014/518261 PMC3976778 24757285

[19] Jin S (2012). Role of p53 in anticancer drug treatment- and radiation-induced injury in normal small intestine. Cancer Biol Med.

[20] Kim W, Lee S, Seo D, Kim D, Kim K, Kim E, Kang J, Seong KM, Youn H, Youn B (2019). Cellular stress responses in radiotherapy. Cells.

[21] Alessenko AV, Shupik MA, Gutner UA, Bugrova AE, Dudnik LB, Shingarova LN, Mikoyan A, Vanin AF (2005). The relationship between sphingomyelinase activity, lipid peroxidation and NO-releasing in mice liver and brain. FEBS Lett.

[22] Yahyapour R, Amini P, Rezapour S, Cheki M, Rezaeyan A, Farhood B, Shabeeb D, Musa AE, Fallah H, Najafi M (2018). Radiation-induced inflammation and autoimmune disease. Mil Med Res.

[23] Lei Y, Wang K, Deng L, Chen Y, Nice EC, Huang C (2015). Redox regulation of inflammation: old elements, a new story. Med Res Rev.

[24] Abias JM (2015). Redox regulation of NLRP3 inflammasomes: ROS as trigger or effector?. Antioxid Redox Signal.

[25] Wei J et al (2019) Radiation-induced normal tissue damage: oxidative stress and epigenetic mechanisms. Oxid Med Cell Longev 2019:3010342 10.1155/2019/3010342 PMC6875293 31781332

[26] Azzam EI, Jay-Gerin JP, Pain D (2012). Ionizing radiation-induced metabolic oxidative stress and prolonged cell injury. Cancer Lett.

[27] Ighodaro OM, Akinloye OA (2018) First line defence antioxidants – superoxide dismutase (SOD), catalase (CAT) and glutathione peroxidase (GPX): Their fundamental role in the entire antioxidant defence grid. Alex J Med 54:287–293

[28] Wang Y, Branicky R, Noë A, Hekimi S (2018). Superoxide dismutases: dual roles in controlling ROS damage and regulating ROS signaling. J Cell Biol.

[29] Candas D, Li JJ (2014). MnSOD in oxidative stress response-protected regulation via mitochondrial protein influx. Antioxid Redox Signal.

[30] Holley AK, Miao L, St. Clair DK, St. Clair WH (2014). Redox-modulated phenomena and radiation therapy. The central role of superoxide dismutases. Antioxid Redox Signal.

[31] Miao L, St Clair DK (2009). Regulation of superoxide dismutase genes: Implications in diseases. Free Radic Biol Med.

[32] Guo (2003). Manganese superoxide dismutase-mediated gene expression in radiation injury adaptive responses. Mol Cell Biol.

[33] Culetta VC (2006). Activation of superoxide dismutase: putting the metal to the pedal. Biochim Biophys Acta.

[34] Morgan MJ, Liu Z (2011). Crosstalk of reactive oxygen species and NF-κB signaling. Cell Res.

[35] Woo Y et al (2019) mTOR-mediated antioxidant activation in solid tumor radioresistance. J Oncol 2019:5956867. 10.1155/2019/5956867 10.1155/2019/5956867 PMC6942807 31929797

[36] Zhou R, Yazdi AS, Menu P, Tschopp J (2011). A role for mitochondria in NLRP3 inflammasome activation. Nature.

[37] Sonis ST (2010). New thoughts on the initiation of mucositis. Oral Dis.

[38] Wei J, Wang H, Wang H, Wang B, Meng L, Xin Y, Jiang X (2019). The role of the NLRP3 inflammasome activation in radiation damage. Biomed Pharmacother.

[39] Abderrazak A, Syrovets T, Couchie D, el Hadri K, Friguet B, Simmet T, Rouis M (2015). NLRP3 inflammasome: from a danger signal sensor to a regulatory node of oxidative stress and inflammatory disease. Redox Biol.

[40] Hoesel B, Schmid JA (2013). The complexity of NF-κB signaling in inflammation and cancer. Mol Cancer.

[41] Holley AK, Xu Y, Clair DKS, Clair WHS (2010). RelB regulates manganese superoxide dismutase gene and resistance to ionizing radiation of prostate cancer cells. Ann N Y Acad Sci.

[42] Sies H (2017). Hydrogen peroxide as a central redox signaling molecule in physiological oxidative stress: oxidative eustress. Redox Biol.

[43] Oliveira-Marques V (2009). Role of hydrogen peroxide in NF-κB activation: from inducer to modulator. Antioxid Redox Signal.

[44] Lee C, Blum JM, Kirsch DG (2013). Role of p53 in regulating tissue response to radiation by mechanisms independent of apoptosis. Transl Cancer Res.

[45] Mortezaec K (2019). NF-κB targeting for overcoming tumor resistance and normal tissue toxicity. J Cell Physiol.

[46] Normando AGC, Rocha CL, de Toledo IP, de Souza Figueiredo PT, dos Reis PED, de Luca Canto G, Guerra ENS (2017). Biomarkers in the assessment of oral mucositis in head and neck cancer patients: a systematic review and meta-analysis. Support Care Cancer.

[47] Pires BRB, Silva R, Ferreira G, Abdelhay E (2018). NF-kappaB: Two sides of the same coin. Genes (Basel).

[48] Pardo M, Tirosh O (2009). Protective signaling effect of manganese superoxide dismutase in hypoxia-reoxygenation of hepatocytes. Free Radic Res.

[49] Nguyen T, Nioi P, Pickett CB (2009). The Nrf2-antioxidant response element signaling pathway and its activation by oxidative stress. J Biol Chem.

[50] Moldogazieva NT et al (2019) Oxidative stress and advanced lipoxidation and glycation end products (ALEs and AGEs) in aging and age-related diseases. Oxidative Med Cell Longev 2019:3085756. 10.1155/2019/3085756 10.1155/2019/3085756 PMC6710759 31485289

[51] Pekovic-Vaughan V (2014). The circadian clock regulates rhythmic activation of the Nrf2/glutathione mediated antioxidant defense pathway to modulate pulmonary fibrosis. Genes Dev.

[52] Goyal M (2009). Oral mucositis in morning vs. evening irradiated patients: a randomized prospective study. Int J Radiat Biol.

[53] Cameron BD (2018). The role of Nrf2 in the response to normal tissue radiation injury. Radiat Res.

[54] Anmuranjani MB (2014). Concerted action of Nrf2-ARE pathway. Redox Biol.

[55] Wang P, Li CG, Qi Z, Cui D, Ding S (2016). Acute exercise stress promotes Ref1/Nrf2 signaling and increases mitochondrial activity in skeletal muscle. Exp Physiol.

[56] Wardyn JD, Ponsford AH, Sanderson CM (2015). Dissecting molecular cross-talk between Nrf2 and NF-κB response pathways. Biochem Soc Trans.

[57] Banning A (2005). NF-κB, Nrf2, and HO-1 interplay in redox-regulated VCAM expression. Antioxid Redox Signal.

[58] Pishochi AM, Pop A (2015). The role of antioxidants in the chemistry of oxidative stress: a review. Eur J Med Chem.

[59] Maier P, Hartmann L, Wenz F, Herskind C (2016). Cellular pathways in responses to ionizing radiation and their targetability for tumor radiosensitization. Int J Mol Sci.

[60] Rodemann HP, Blases MA (2007). Responses of normal cells to ionizing radiation. Semin Radiat Oncol.

[61] Kim R, Emi M, Tanabe K (2005). Caspase-dependent and independent cell death pathways after DNA damage (Review). Oncol Rep.

[62] Hoye AT, Davoren JE, Wipf P, Fink MP, Kagan VE (2008). Targeting mitochondria. Acc Chem Res.

[63] Lee C (2013). Role of p53 in regulating tissue response to radiation independent of apoptosis. Transl Cancer Res.

[64] Drane P, Bravard A, Bouvard V, May E (2001). Reciprocal down regulation of p53 and SOD2 gene expression – implication in p53-mediated apoptosis. Oncogene.

[65] Azzam EI, Little JB (2004). The radiation-induced bystander effect: evidence and significance. Hum Exp Toxicol.

[66] King M, Joseph S, Albert A, Thomas TV, Nittala MR, Woods WC, Vijayakumar S, Packianathan S (2020). Use of amifostine for cytoprotection during radiation therapy: a review. Oncology.

[67] Henke M, Alfonsi M, Foa P, Giralt J, Bardet E, Cerezo L, Salzwimmer M, Lizambri R, Emmerson L, Chen MG, Berger D (2011). Palifermin decreases severe mucositis undergoing postoperative radiochemotherapy for head and neck cancer; a randomized, placebo-controlled trial. J Clin Oncol.

[68] Le QT (2011). Palifermin reduces severe mucositis in definitive chemoradiotherapy of locally advanced head and neck cancer: a randomized, placebo-controlled study. J Clin Oncol.

[69] Blijlevens N, Sonis S (2007). Palifermin (Recombinant keratinocyte growth factor-1): a pleotropic growth factor with multiple biological activities in preventing chemotherapy- and radiotherapy-induced mucositis. Ann Oncol.

[70] Rosenthal RA (2011). Salen Mn complexes mitigate radiation injury in normal tissues. Anti Cancer Agents Med Chem.

[71] Murphy CK, Fey EG, Watkins BA, Wong V, Rothstein D, Sonis ST (2008). Efficacy of superoxide dismutase mimetic M40403 in attenuating radiation-induced oral mucositis in hamsters. Clin Cancer Res.

[72] Petkau A (1987). Role of superoxide dismutase in modification of radiation injury. Br J Cancer.

[73] Escribano A, García-Grande A, Montañés P, Miralles L, García A (2002). Aerosol orgotein (Ontosein) for the prevention of radiotherapy-induced adverse effects in head and neck cancer patients: a feasibility study. Neoplasma.

[74] Esco R, Valencia J, Coronel P, Carceller JA, Gimeno M, Bascón N (2004). Efficacy of orgotein in prevention of late side effects of pelvic irradiation: a randomized study. Int J Radiat Oncol Biol Phys.

[75] Epperly MW, Kagan VE, Sikora CA, Gretton JE, Defilippi SJ, Bar-Sagi D, Greenberger JS (2001). Manganese superoxide dismutase plasmid/liposome (MnSOD-PL) administration protects mice from esophagitis with fractionated radiation. Int J Cancer.

[76] Anderson C (2019). Phase IIb, randomized, double-blind trial of GC4419 versus placebo to reduce severe oral mucositis due to concurrent radiotherapy and cisplatin for head and neck cancer. J Clin Oncol.

[77] Ara G, Watkins BA, Zhong H, Hawthorne TR, Karkaria CE, Sonis ST, Larochelle WJ (2008). Velafermin (rhFGF-21) reduces the severity and duration of hamster cheek pouch mucositis induced by fractionated radiation. Int J Radiat Biol.

[78] Reisman SA, Lee CYI, Meyer CJ, Proksch JW, Sonis ST, Ward KW (2014). Topical application of the synthetic triterpenoid RTA 408 protects mice from radiation-induced dermatitis. Radiat Res.

[79] Yerra VG (2013). Potential therapeutic effects of simultaneous targeting of Nrf2 and NF-κB pathways in diabetic neuropathy. Redox Biol.

[80] Oronsky B, Goyal S, Kim MM, Cabrales P, Lybeck M, Caroen S, Oronsky N, Burbano E, Carter C, Oronsky A (2018). A review of clinical radioprotection and chemoprotection of oral mucositis. Transl Oncol.

[81] Giralt J, Tao Y, Kortmann RD, Zasadny X, Contreras-Martinez J, Ceruse P, Arias de la Vega F, Lalla RV, Ozsahin EM, Pajkos G, Mazar A, Attali P, Bossi P, Vasseur B, Sonis S, Henke M, Bensadoun RJ (2020). Randomized phase 2 trial of a novel clonidine mucoadhesive buccal tablet for the amelioration of oral mucositis in patients treated with concomitant chemo-radiotherapy for head and neck cancer. Int J Radiat Oncol Biol Phys.

[82] Kudrimoti M, Curtis A, Azawi S, Worden F, Katz S, Adkins D, Bonomi M, Elder J, Sonis ST, Straube R, Donini O (2016). Desquetide: a novel innate defense regulator demonstrating a significant and consistent reduction in the duration of oral mucositis in preclinical data and a randomized, placebo-controlled Phase 2a clinical study. J Biotechnol.

